# Tooth Loss in Periodontitis Patients—A Risk Factor for Mild Cognitive Impairment: A Systematic Review and Meta—Analysis

**DOI:** 10.3390/jpm14090953

**Published:** 2024-09-09

**Authors:** Bhawna Agarwal, Maria Eleonora Bizzoca, Gennaro Musella, Danila De Vito, Lorenzo Lo Muzio, Andrea Ballini, Stefania Cantore, Flavio Pisani

**Affiliations:** 1 School of Medicine and Dentistry, University of Central Lancashire, Preston PR1 2HE, UK; bhawna30a@gmail.com (B.A.); fpisani@uclan.ac.uk (F.P.); 2 Department of Clinical and Experimental Medicine, University of Foggia, Via Rovelli 50, 71122 Foggia, Italy; mariaeleonora.bizzoca@unifg.it (M.E.B.); gennaro.musella@unifg.it (G.M.); lorenzo.lomuzio@unifg.it (L.L.M.); andrea.ballini@unifg.it (A.B.); 3 School of Medicine, University of Bari, 70100 Bari, Italy; danila.devito@uniba.it; 4 Department of Precision Medicine, University of Campania Luigi Vanvitelli, Via De Crecchio, 7, 80138 Naples, Italy

**Keywords:** tooth loss, periodontitis, mild cognitive impairment

## Abstract

Background: Periodontal disease and tooth loss have been long suggested as risk factors of mild cognitive impairment. The underlying mechanisms could be systemic chronic inflammatory mediators, direct pathologic challenge to the nervous system, malnutrition and/or loss of neurosensory stimulation input causing brain atrophy. This review aimed to examine the existing literature studies linking the effect of periodontal disease and tooth loss on the development of mild cognitive impairment. Methods: A systematic review using PEO was conducted. Three electronic databases, namely Embase, Medline and DOSS (UCLan), were searched for relevant articles published up to April 2023. Google Scholar and a hand search were also conducted to ensure no relevant studies had been missed. The Newcastle–Ottawa scale was used to assess the quality of studies. Results: The findings showed that chronic periodontitis and tooth loss, both individually and in combination, led to an increased risk of mild cognitive decline in adults over 50 years. Within the limitations of this review, periodontitis and tooth loss both contribute to an increased risk of mild cognitive impairment and dementia, but the evidence so far is not strong. Conclusions: In future, more robustly designed studies investigating periodontal disease and tooth losslink with cognitive health decline are required with a longer follow-up duration.

## 1. Introduction

Dementia is a common, non-communicable syndrome affecting elderly people and is recognized by irreversible cognitive deterioration and functional decline, including symptoms such as memory loss, confusion, inability to perform day to day routine jobs, behavioral changes, and social and functional incapacitation. To date, there is no effective drug or treatment to cure dementia [1]. Along with other neurodegenerative disorders such as Alzheimer’s disease (AD), dementia causes a massive impairment of quality of life and contributes to huge financial burden on the social and economic infrastructure and health care bodies globally [2,3]. Worldwide, over 50 million people are affected by dementia, and the number is expected to increase up to three times by 2050 [3]. In about one-fifth of dementia cases, including Alzheimer’s disease, vascular dementia and other types, mild cognitive impairment (MCI) has been shown as a symptom in the preceding 5 years [4]. MCI is characterized by cognitive decline which is greater than expected for the normal person of a certain education level and age but does not interfere with usual day to day activities or quality of life or, in other words, functional daily activities [5]. The pathological features of MCI and dementia are the same as per the *Diagnostic and Statistical Manual of Mental Disorders* (DSM-5), which explains the high number of MCI cases progressing to dementia [6,7,8]. A study of epidemiological evidence elicited multiple risk factors for dementia and MCI, including smoking, unhealthy diet such as low vegetable consumption and high saturated fat consumption, low levels of education and a less active lifestyle mentally and socially. Medical conditions such as cardiovascular diseases, high blood pressure, genetic predisposition (APOE e4) and diabetes mellitus have also been shown to contribute to a risk of developing cognitive decline [9,10,11]. The role of inflammation affecting the central nervous system causing neuronal damage has also been suggested as a contributing factor for dementia and mild cognitive decline [12]. A connection between tooth loss due to periodontal diseases, on one hand, and dementia and Alzheimer’s disease, on the other hand, has been studied with interest over many years. Supporting this association, a study on twins elicited strong links between Alzheimer’s disease and the number of teeth lost [13]. Supporting these findings, another study demonstrated that a higher number of functional teeth and functional occlusal units were related to lower odds of cognitive impairment [14]. As many long-term longitudinal studies have shown that the outcome of uncontrolled periodontal disease is tooth loss [15,16], this suggests there is a link among periodontitis, tooth loss, Alzheimer’s disease and dementia [17]. Periodontitis is a chronic inflammatory disease that leads to progressive irreversible destruction of the supporting tissues around teeth, which, if uncontrolled, may result in premature tooth loss [18,19]. According to the Global Burden of Disease 2015 study, severe forms of periodontal disease affected 7.4% of the population worldwide [20], whereas milder forms were prevalent in as much as 50% of people [21,22]. Tooth loss because of periodontitis and dental caries has imposed a huge burden and years of disability, higher than any other human illness [23]. On the basis of the resulting systemic inflammatory burden, strong links have also been established between periodontal diseases and systemic conditions such as diabetes, cardiovascular diseases and adverse pregnancy outcomes (as low birth weight or miscarriage) [21].

Various possible mechanisms linking the association of periodontal disease with dementia have been investigated [24,25]. Bacterial endotoxins as lipopolysaccharides (LPS) and pro-inflammatory products such as C-reactive protein (CRP), tumor necrosis factor-alpha (TNF-alpha), and interleukin-1 and -6 (IL-1, -6) resulting from periodontal disease have been suggested to increase the risk of nervous system inflammation entering via the oral cavity [17]. A direct migration of pathogens such as *Porphyromonas gingivalis* along the nerves from inflammatory lesions in the mouth to cranial structures has also been implicated as a possible route of infection [26]. Other studies have assessed the impact of tooth loss as a modifiable risk factor for dementia and MCI [27,28,29]. Saying this, only a few longitudinal studies exist where a causal association between loss of teeth and memory impairment or cognitive decline has been investigated [30,31,32]. Tonsekar et al. (2017) conducted a systematic review on the association of periodontal disease, tooth loss and dementia, and concluded that although there is an association, the evidence is too weak to show that chronic periodontal disease and tooth loss are risk factors for dementia [33]. Another recent systematic review aiming to establish evidence of a causative association of chronic periodontitis and cognitive decline found that poor periodontal health and tooth loss both seem to increase risk of cognitive impairment and dementia; however, it did not address whether the tooth loss was a result of periodontal disease [34]. Another shortcoming in the aforementioned review was that some of the studies that were included to assess the risk of mild cognitive decline included self-reported tooth loss which was not clinically examined. A few other authors have also reached the conclusion that the overall evidence available to prove a causal association among periodontitis, tooth loss and MCI is still insufficient and further analysis is required in this field [35,36].

Considering the high worldwide prevalence of periodontal disease and tooth loss, as well as mild cognitive impairment [2,37], this systematic review of literature studies aimed to fill the gap and to investigate if there is evidence of an increased risk of mild cognitive impairment resulting from tooth loss because of periodontitis in older adults. An increased awareness and evidence of periodontitis and related tooth loss, as risk factors of mild cognitive impairment, will help to develop future public health policies and implement interventions to improve the oral health of the masses and delay or prevent the onset of Alzheimer’s disease and dementia, which are considered irremediable so far [38].

This systematic review aimed to fill the evidence gap of a causative link between chronic periodontal diseases, the corresponding tooth loss and the risk of developing mild cognitive decline in older adults aged 50 years and above.

The objective was to search the existing literature for studies that have investigated an association of periodontitis, tooth loss and cognitive decline, and to find out the risk of a causative association through data extraction and analysis.

## 2. Materials and Methods

PEO was used to frame the research question. The population (P) was healthy adults with no signs of cognitive impairment and dementia at baseline, the exposure (E) was periodontal disease and tooth loss, and the outcome (O) was mild cognitive impairment (MCI)/cognitive decline. On the basis of the existing literature, this review aimed to answer the following question: “Is there an increased risk of developing mild cognitive impairment in older adults as a result of being exposed to infection and tooth loss secondary to periodontitis?”

### 2.1. Methods

This systematic review of literature studies was conducted as per the principles outlined in Preferred Reporting Items for Systematic Reviews and Meta-analysis (PRISMA) [39], following a rigorous methodology to minimize the risk of bias using a PEO approach. This study has been registered in PROSPERO (CRD42024576439).

For the review process, periodontal disease was defined as an inflammatory condition resulting in progressive destruction of tooth-supporting tissues characterized by clinical attachment level loss (CAL loss), the presence of deep pockets (PPD), bleeding on probing (BoP) and bone loss on radiographs (ABL) [40], which, if left untreated, could result in premature tooth loss [37]. As uncontrolled periodontitis can result in accelerated tooth loss, a low tooth count was taken as a representation of a history of long-term periodontal disease. Nevertheless, teeth can also be lost due to other causes, and this was considered as an exclusion criterion.

A recognition of cognitive impairment or memory impairment in individuals as compared with other people of the same age, without any deterioration in functional abilities and no dementia has been recognized as the subjective definition of MCI and supported by objective measures such as a deficit in cognitive abilities through validated batteries of neuropsychological tests. Usually, executive functions, attention, language, memory and visuospatial skills are considered [41]. Our hypothesis was that the inflammatory process related to periodontal disease may influence the pathogenesis of Alzheimer’s disease by worsening dementia, its main manifestation, in a synergic way.

### 2.2. Search Design

A systematic search for human studies, restricted to the English language, was conducted in three electronic databases: MEDLINE, Embase and Dentistry/Oral Sciences source (DOSS via UCLan) on the EBSCO host platform up to April 2023. An additional search was carried out on Google Scholar using free text, which included periodontitis, periodontal disease, tooth loss and mild cognitive impairment, and a hand search were performed, using the references of the cited articles to include all the relevant articles. Keywords using Boolean operators and MeSH headings were loss of teeth, tooth loss (MeSH), chronic periodontitis (MeSH), periodontal disease, periodontitis, chronic periodontal diseases, mild cognitive impairment (MeSH), mild cognitive decline, cognitive deterioration, mental deterioration, mild neurocognitive disorder, mild memory loss, mild memory impairment and mild amnesia. At this stage, observational studies conducted on humans, which investigated an association between cognitive decline/brain function verified by neuropsychological tests and periodontitis and tooth loss as confirmed by clinical examination were identified for further screening.

### 2.3. Inclusion Criteria

- Longitudinal prospective cohort and cross-sectional human studies published up to and including April 2023, with limitations to the English language.
- The exposure of interest were chronic periodontal disease and tooth loss, ensuring that at least one of these or both had been assessed and recorded by means of a clinical examination by a qualified dental professional.
- The outcome of interest was individuals diagnosed with mild cognitive deterioration through verified tests such as the mini-mental state examination (MMSE), delayed word recall (DWR) and the digit symbol substitution test (DSST). No restrictions were imposed on the age, gender, socioeconomic status or ethnic status of the subjects.

### 2.4. Exclusion Criteria

- Studies which assessed the effect of dementia or Alzheimer’s disease on overall dental health were excluded from this review.
- Those studies where the outcome differed from mild cognitive impairment or where the exposure factors were other than periodontitis and tooth loss, were not included in this review (such as smoking, diabetes and other risk factors common to both diseases). In this case, we could consider these as hidden confounding factors and hence a limitation for this review. Additionally, studies where information was gathered by means other than clinical examinations, such as surveys or interviews, were also excluded.
- Case–control studies, case reports, reviews and animal studies were excluded.

### 2.5. Selection Process

**Figure 1:**
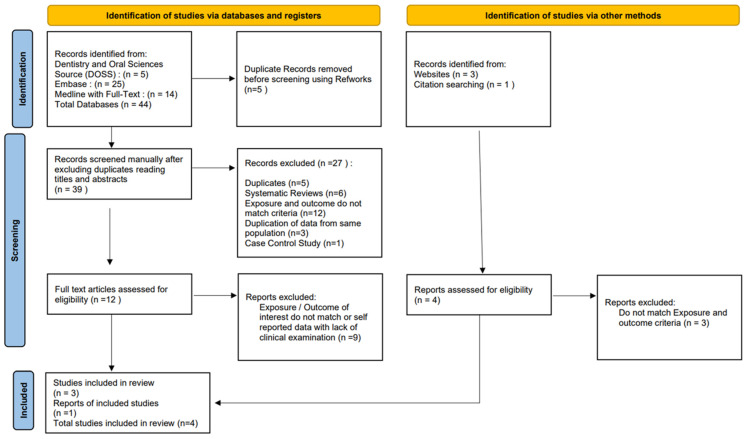
PRISMA flow diagram outlining the process of selecting studies [39].

After the initial search, all the retrieved search results were imported to Reference Manager software (RefWorks ver. 4.6.241), and duplicates were marked and eliminated. Three levels of screening were performed on the titles, abstracts and fully retrieved articles. Two reviewers were involved (BA and FP) and a third one clarified any disagreement (MK). An inter-agreement score was calculated at each stage (Cohen’s K score) [42]. Where multiple studies addressed the same set of population or data, the study with the most raw data was selected for further eligibility testing. The strategy followed for shortlisting the final articles included in this review and the elimination criteria can be explained by the Preferred Reporting Items for Systematic Reviews and Meta-Analyses (PRISMA) flow diagram [39] to ensure a rigorous and transparent approach to the process (Figure 1).

### 2.6. Data Extraction

A customized data extraction form in accordance with the guidance of the *Cochrane Handbook for Systematic Reviews of Interventions* [42] was used for the purpose of this review, and the following information was retrieved and tabulated for all the selected studies (Table 1).

1. General information: The study’s title, authors, year of publication, publisher/source of study and study design;
2. Population and setting: Description of the population, location of the study and the method of recruiting subjects;
3. Methods: Aim of the study, date of the start of data collection and the end date, total duration of observations, ethical approval obtained, and written consent obtained from participants;
4. Participants: Total number of participants at baseline, withdrawals and dropouts by the time of follow up, age at baseline recruitment and gender;
5. Exposure: Methods to evaluate periodontal health such as measurements of probing depth and alveolar bone height in radiographs, and the measures used to assess the extent of severity of disease; the number of remaining teeth at baseline were counted and then again at follow up; and the number of missing teeth were measured as the difference between the two readings;
6. Outcome: Tests to assess cognitive function;
7. Conclusion: A summary of conclusions as derived by the authors from individual studies;
8. Others: Funding sources and conflicts of interest.

### 2.7. Assessment of Quality 

An assessment of the quality of all quantitative studies was performed using the Newcastle–Ottawa Quality assessment scale for cohort studies [41] within eight items under three domains, according to the AHRQ standards (Table 2).

- Good/high quality: 3 or 4 stars in the selection domain AND 1 or 2 stars in the comparability domain AND 2 or 3 stars in the outcome/exposure domain;
- Fair/moderate quality: 2 stars in the selection domain AND 1 or 2 stars in the comparability domain AND 2 or 3 stars in the outcome/exposure domain.
- Low/poor quality: 0 or 1 star in the selection domain OR 0 stars in the comparability domain OR 0 or 1 stars in the outcome/exposure domain [41].

### 2.8. Quantitative Analysis

In addition to a descriptive synthesis, an attempt was also made to carry out a subgroup meta-analysis using Review Manager 5.3 software in accordance with the *Cochrane Handbook for Systematic Reviews of Interventions* [41], to collate quantitative data from studies and derive a numerical estimate of the overall effect of interest. A generic inverse variance method was used. As the selected studies had methodological variations and the effects were reported by the authors, after different models had been adjusted for multiple confounding factors such as medical conditions, access to health insurance etc., values pertaining to crude associations between the exposure and outcome variables were considered for the purpose of the meta-analysis. The most widely used effect estimate, that is, the odds ratio (OR), was used to compute fixed-effects pooled estimates with the 95% confidence interval (CI). When different classes of tooth loss and the severity of periodontal disease were recorded, the effects describing the highest disease scores were used for analysis, as described earlier in the literature [28]. With regards to cognitive decline, estimates relating to more comprehensive and validated tests such as MMSE were used.

Statistical heterogeneity among the studies was assessed using I^2^ test with a level of significance of *p* < 0.01. The I^2^ test measures the heterogeneity between studies based on actual variation and not depending on chance [42,47], and it can vary between 0% and 100%. The weight each study contributed with was reported in a Forrest plot for all three analyzed associations. 

## 3. Results

The results of searching the electronic databases yielded a total of 44 studies. These were imported to Reference Manager software (ver. 4.6.241, and duplicate studies were eliminated using automation tools. From the resulting 39 studies, the titles and abstracts were screened and 12 articles were picked up for full-text (reading) review. In addition, four more articles were found via Google Scholar and by hand searching through the cited references of the selected twelve studies (Figure 1). Discarded articles from the second and third rounds of screening were meticulously recorded with reasons for rejection (Table 3).

After appraising the full texts, four studies were included in this review. The kappa score (K score = 0.83) showed substantial inter-agreement between the reviewers throughout the whole screening process.

Table 1 outlines a summary of the characteristics of all the four selected studies. Out of the four, three studies had a prospective cohort design [43,44,45], and one was cross-sectional [46]. In total 12,079 participants were recruited at baseline across three countries (Sweden, United States and Japan). According to the Newcastle–Ottawa scale scoring, one out of the total four studies was deemed to be of low quality [44], one as fair or moderate quality [46], and two as good/high quality [43,45] (Table 2).

In most of the studies, the age of the participants was over 60 years at baseline, but in one of them [43], even younger males ranging from 24 years onwards were included. Periodontal disease and tooth loss were considered as the exposure of interest, and all the studies examined the oral health status of the participants via clinical examinations with or without a supplementary radiographic investigation, which was performed by qualified calibrated professionals such as dentists, hygienists and periodontists. All of them recorded the number of teeth present at baseline and the loss of teeth as an exposure factor; however, only two included fully erupted third molars in the count [44,46]. Different studies assessed the loss of teeth over the number of years in different ways. Kaye et al. (2010) [43] evaluated number of teeth lost per decade, whereas Okamoto et al. (2015) [44] noted a reduction in the ranking of the categories of remaining number of teeth at follow-up in five groups, namely 0/1–8/9–16/17–24/25–32, and compared them with the outcomes. Luo et al. (2022) [45] declared significant tooth loss (STL) if eight or more teeth were lost at the follow-up examination compared with the baseline. The majority of studies investigated the severity and progression of periodontal disease by recording the number and proportion of teeth with increasing probing depth measurements, such as over 4 mm, and worsening alveolar bone loss and periodontitis were diagnosed if these sites were ≥30% of the total teeth; however, one of the studies’ utilized the community periodontal index (CPI) as the measure of interest, utilizing ten representative teeth in six segments of the mouth, and the highest code from all the six sextants for that individual was recorded as their maximum score. A significant variation was also noted among authors in defining periodontitis, such as the proportion of teeth exhibiting a reduction in bone height in radiographs by 40% or more at follow-up appointments compared with the baseline [43,46], in contrast to Luo et al. (2022) [45], who employed the criteria of the Centers for Disease Control and Prevention and the American Academy of Periodontology (CDC-AAP) to define chronic periodontal disease as pockets in excess of 4 mm, as well as alveolar bone loss of a minimum of 3 mm [54], and graded the disease on the basis of the severity of interproximal attachment loss and alveolar bone loss as follows: mild, ≥2 interproximal sites with attachment loss of ≥3 mm and ≥2 sites with probing depths of ≥4 mm (not on the same tooth) or ≥1 site with a probing depth of ≥5 mm; moderate, ≥2 interproximal sites with attachment loss of ≥4 mm (not on the same tooth) or ≥2 interproximal sites with a pocket depth of ≥5 mm (not on the same tooth); severe, ≥2 interproximal sites with attachment loss of ≥6 mm (not on the same tooth) and ≥1 interproximal sites with probing depths of ≥5 mm (not on the same tooth).

The definition of cognitive outcomes also varied among the study authors. Three out of the four studies measured mild cognitive impairment (MCI) as an outcome of interest, in contrast to one where the authors kept mild memory impairment (MMI) as the outcome of choice rather than cognitive impairment, with an argument that it would help in eliminating a chance of reverse causality, as cognitive impairment in adults can lead to a decline in oral health. Nevertheless, the definition used to describe MMI was similar to that for MCI [44]. The diagnosis of cognitive decline was mostly assessed using validated tests such as the mini-mental state examination (MMSE), which is recognized and validated and consists of 20 items, and a maximum score of 30 is achievable in healthy people [55]. A diagnosis of a MMSE score of 21–25 is indicative of mild cognitive impairment, which was the outcome of interest. The outcome of an MMI diagnosis was made if the MMSE score was 24 or more, along with a score of 0 or 1 in the word recall test [44]. Other cognitive function tests employed by the studies included in this review included spatial copying tests (SCT), clock tests, six-items screening, word recall tests, digit symbol tests, etc., all of which are validated methods of evaluating cognitive function [8].

### 3.1. Data Synthesis

A descriptive synthesis revealed that, in general, all the studies reported and indicated an increased risk of an association of periodontal disease, tooth loss and old age with cognitive decline. Kaye et al. (2010) [43] estimated that the risk of lower scores for cognitive function increased independently both with the progression of periodontal disease and the number of teeth lost. They estimated adjusted hazards ratio (HR) for each tooth lost over a period of 10 years, with 95% confidence intervals. The time to event was variable from 20–32 years, which was defined as the time interval between the start of the longitudinal dental study and the occurrence of the lowest cognitive score. For each tooth lost over a period of 10 years, the risk of a low MMSE score (<25) and a low SCT score (<10 points) increased by 9–12% (HR = 1.09, 95% CI 1.01, 1.18 and HR = 1.12, 95% CI 1.05, 1.18, respectively). An elevated risk of mild cognitive impairment was also associated with progressive loss of bone height per tooth and both increasing probing depth and low MMSE (HR 1.03, 95% CI 1.00, 1.07 and HR = 1.04, 95% CI 1.01, 1.09, respectively). Table 4 presents the adjusted ratios for age, gender and education with confidence intervals from all four studies for the risk of cognitive decline in relation to the severity of periodontal disease and its progression. The pooled results show that the periodontal disease’s progression was associated with an increased risk of cognitive deterioration; however, the confidence intervals were wide. Overall, 10–46% of men scoring 26 or higher on the MMSE at baseline fell below the cut-off point on repeat testing (MMSE < 25) secondary to tooth loss [43]. Similar observations were made in two other studies, where the loss of eight or more teeth significantly affected the increased incidence of cognitive decline [45], with a cumulative incidence of 11.5% of the population with 17–24 teeth remaining as compared with participants with 25–32 teeth. The odds ratio for the outcome of mild memory impairment in the group with 17–24 remaining teeth and the edentulous group were OR = 1.57 (95% CI, 1.11–2.23, *p* = 0.011) and OR = 2.32 (95% CI, 1.44–3.746, *p* = 0.001), respectively as compared with the subset with 25 to 32 remaining teeth [44]. Table 5 shows the age-, gender- and education-adjusted ratios for the risk of cognitive decline in relation to tooth loss. The findings suggested a strong association between tooth loss and a heightened risk of cognitive decline. Looking into periodontal disease as an exposure, the prevalence of bone loss of 4 mm or more from the cementoenamel junction (CEJ) to marginal bone levels on ≥30% of sites also correlated strongly with low MMSE scores (OR= 2.7, 95% CI 1.2–5.9, *p* = 0.13) [46], in contrast to Okamoto et al. (2015) [44], who could not identify any statistically significant relationship between the community periodontal index codes’ severity and the development of MMI (OR = 1.03, 95% CI 0.73–1.45, *p* = 0.871).

### 3.2. Meta-Analysis

**Figure 2:**
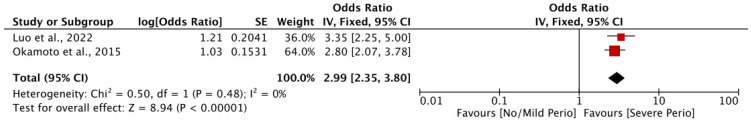
Meta-analysis demonstrating the influence of periodontal disease in two studies [44,45] on the incidence of mild cognitive impairment. Data are presented as pooled odds ratios and 95% CI (horizontal lines). Red square: pooled odds ratio and Confidence intervals (CI) for each study; Black diamond: Overall Pooled odds ratio and CI for the total.

**Figure 3:**
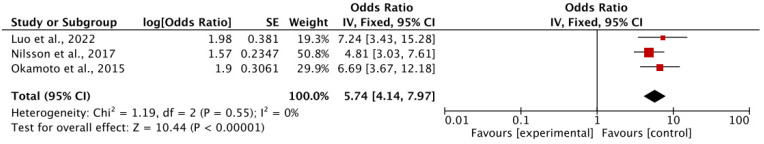
Meta-analysis demonstrating the influence of tooth loss in three studies [45,46,47] on the incidence of mild cognitive impairment. Data are presented as polled odds ratios and 95% CI (horizontal lines). Red square: pooled odds ratio and Confidence intervals (CI) for each study; Black diamond: Overall Pooled odds ratio and CI for the total.

**Figure 4:**
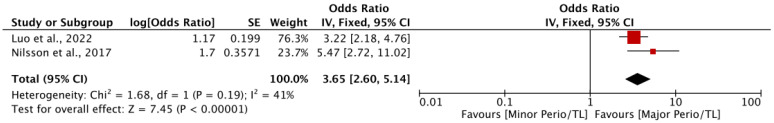
Meta-analyses demonstrating the influence of tooth loss and periodontal disease in two studies [45,46] on the incidence of mild cognitive impairment. Data are presented as polled odds ratios and 95% CI (horizontal lines). Red square: pooled odds ratio and Confidence intervals (CI) for each study; Black diamond: Overall Pooled odds ratio and CI for the total.

The four studies employed different effect estimates, and hence the data were statistically incomparable using all the available estimates and all the studies; however, an attempt was made to carry out a subgroup meta-analysis (Figure 2, Figure 3 and Figure 4) using Review Manager 5.3 software in accordance with *Cochrane Handbook for Systematic Reviews of Interventions* [42]. Multiple meta-analysis models were generated to evaluate different measures of decline in oral health compared with cognitive decline outcomes. Pooled odds ratios (OR) were employed to compute fixed-effects estimates with 95% confidence intervals. The effects describing the exposure as the highest disease scores were used for the analysis, as was described earlier in the literature [28]. With regards to cognitive decline, estimates relating to more comprehensive and validated tests such as MMSE were used. Heterogeneity was measured among the studies using the Chi-squared test (I^2^). As Kaye et al. (2010) [43], measured and reported hazards ratio estimates, unlike the others, it was not possible to include their data in the meta-analysis.

#### 3.2.1. Association of Periodontal Disease and Cognitive Decline

The results from the meta-analysis of two studies demonstrated that periodontal disease (Figure 2) was associated with a higher risk of developing MCI. The most severe forms of periodontitis, as measured by CPI Code 4 was strongly related to a higher risk of cognitive decline (pooled OR = 2.99; 95% CI 2.35–3.80; *p* < 0.00001), with the highest degree of homogeneity [44,45]. In this case, the study by Okamoto et al. [44], contributed the most (64.0%).

#### 3.2.2. Association of Tooth Loss and Cognitive Decline

Figure 3 shows that the loss of teeth was independently associated with a high risk of developing MCI or cognitive deterioration over time, with a pooled odds ratio of 5.74 but with a wide 95% CI (4.14, 7.97) in the three studies [44,45,46]. In the aforementioned analysis, the studies were found to be homogenous. (I^2^ = 0). In this association analysis, the study by Nilsson et al. [46], contributed the most (50.8%), while the other two studies were found to contribute quite equally (Luo et al. [45]: 19.3% and Okamoto et al. [44]: 29.9%).

#### 3.2.3. Association of Periodontal Disease and Tooth Loss Together with Cognitive Decline

The pooled odds ratios for periodontal disease and tooth loss as combined exposure factors showed similar results (OR = 3.65; 95% CI 2.60–5.14; *p* < 0.00001), again with a high degree of homogeneity (I^2^ = 41%) [44,45,46] (Figure 4). From this perspective, almost all the overall weight was provided by Luo et al. [45] (76.3%).

## 4. Discussion

This systematic review aimed to investigate the association among chronic periodontal disease, tooth loss and the risk of developing mild cognitive impairment in older adults. The review followed the guidelines outlined in the Preferred Reporting Items for Systematic Reviews and Meta-Analyses and included studies published up to 14 April 2023. Overall, the findings from the four selected studies suggested that individuals who suffered from long-term periodontal disease and tooth loss presented a higher risk of developing mild cognitive impairment than those who were not affected with periodontal disease or had not lost any teeth. However, due to clinical, methodological and statistical heterogeneity as well as the large sample attrition among the studies, the overall quality of evidence is very low. The association of periodontal disease as a risk towards developing cognitive impairment was statistically insignificant, as demonstrated by the data’s confidence intervals of 0.73–1.45 and 0.67–1.54 found by Okamoto et al. (2015) [44] and Luo et al. (2022) [45], respectively. In contrast, the loss of many teeth or turning edentulous from dentate carried a high risk of an association with cognitive decline, as demonstrated by Okamoto et al. (2015) [44], (OR: 2.32; 95% CI: 1.44, 3.74; *p* = 0.001) and Nilsson et al. (2018) [46], (OR: 2.0; 95% CI: 1.1, 3.6; *p* = 0.01). Loss of individual teeth compared with the baseline, however, did not contribute much to the impairment of cognition (OR: 1.01; 95% CI: 1.0, 1.03; *p* = 0.051).

Only two out of four studies with periodontitis as an exposure showed a significant association with MCI in the findings of the subgroup meta-analysis. Nevertheless, the risk of being prone to the development of mild cognitive decline when exposed to increasing tooth loss was significantly higher and was supported by 75% of the studies: OR = 5.74 at 95% confidence intervals, compared with OR = 2.99 and OR = 3.65 for periodontal disease alone as the exposure and periodontal disease and tooth loss together, respectively. These findings corroborate with the findings of the periodontal risk assessment by Lang and Tonetti, (2003) where a loss of eight or more teeth shifted the disease’s prognosis to a higher risk compared with a moderate risk when four to eight teeth are lost.

The results suggest that apart from the suspected role of chronic inflammatory states created by periodontitis that may cause inflammation in the central nervous system [56], there must be other mechanisms through which the loss of teeth affects brain function and cognition.

The word “cognition” can be used to refer to a group of mental functions performed by the brain, such as memory, logic and reasoning, recognition, judgement, planning and organization, movement coordination (praxis), etc. All these aforementioned abilities are part of basic survival and the interaction strategies of humans and give us an identity in society and a larger meaning to individual life. Therefore, any condition/disease which leads to cognitive deterioration has a big impact on the existence and quality of life for an individual and their family [29]. Various studies have demonstrated that the cerebral cortex in the brain has sites that are stimulated by inputs from the teeth. The mechanoreceptors present in the periodontal ligaments surrounding the teeth play a crucial role in communicating information to the cerebrum from the oral cavity. Chronic periodontal disease leads to loss of the periodontal ligament apparatus around the teeth, thus causing an interruption in the tooth–brain connection and adversely affecting the brain’s processes [57]. However, it is not clear as to how these alterations happen in the brain’s activity secondary to tooth loss and periodontal disease.

Loss of multiple teeth may be connected to the development of cognitive decline in multiple ways. Loss of multiple teeth results in occlusal disharmony and reduced masticatory forces and sensorimotor stimulation from the masticatory apparatus. This, in turn, may lead to reduced cerebral flow and diminished proprioception and reduced blood flow to the brain. MRI (magnetic resonance imaging) studies in humans have proven that masticatory movements activate different brain centers, including the thalamus and cerebellum [58,59]. Similar findings have been reported by other authors too, linking atrophy of the gray matter in brain with tooth loss and the loss of masticatory efficiency [48]. Complementing above studies are the results from J.H. Han et al. (2020) [14], who found that the replacement of lost natural teeth by fixed prostheses and implants, thus replacing functional occlusal units, was inversely related to cognitive impairment [14]. Contrastingly, chewing function with removable dentures results in more than 50% reduced masticatory efficiency and hence does not contribute to maintaining cerebral blood flow [60]. Interestingly several animal studies also support the above findings. A study reported that when the crowns of molar teeth were cut off in rats to eliminate masticatory input, a diminution in the number of choline acetyltransferase-positive neurons was seen in the septal nucleus [61]. In mice with extracted molars, hippocampal changes were noticed, showing a reduction in pyramidal cells [62]. These findings from animal studies support human studies; however, more investigation is warranted.

Additionally, systemic inflammation resulting from periodontal disease causes an increased load of circulating pro-inflammatory cytokines and may facilitate neuroinflammation of the central nervous system. Studies that support this theory have shown that non-steroidal anti-inflammatory drugs slow down the onset of dementia [63]. The formation of amyloid plaques causing vascular damage is also seen in Alzheimer’s disease, which, in turn, is caused by triggered glial cells secreting pro-inflammatory cytokines [26].

The direct migration of pathogenic microflora from the oral cavity to the brain via the channels of the trigeminal nerve cannot be ruled out as a contributing factor. Various species of *Treponemae*, including *T. denticola*, have been recovered from the brain [64]. Endothelial dysfunction has also been investigated as a probable underlying mechanism leading to dementia [65]. Endotoxins such as LPS and other virulence factors from pathogens such as *P. gingivalis*, *A. actinomycetemcomitans* and *T. denticola* can damage the endothelial walls of blood vessels [66].

Another plausible connection between periodontitis and dementia comes from genetic studies. It has been elucidated that the apolipoprotein E (APoE) e4 allele has been interlinked with susceptibility to Alzheimer’s disease and dementia [31,67]. However, none of the studies included in this review assessed the APoE genotype of the participants.

These results, however, should be considered in the context of certain limitations. Firstly, there were clinical and methodological variations in the studies’ design, such as different authors using different methods for assessing cognitive decline, and the extent and severity of periodontal disease, and substantial heterogeneity existed when calculating the number of teeth lost. Secondly, none of the studies considered race as a possible confounding factor, and none reported the main risk factors that might cause either periodontitis or neuroinflammation and degeneration as smoking or Type 1 and 2 diabetes. Thirdly, the studies reported different effect estimates such as hazards ratios and adjusted odds ratios, leading to the low quality of the evidence. Furthermore, the pooled analysis was only possible when considering periodontal probing depths; even though it is an essential indicator of periodontal disease, loss of clinical attachment is a more accurate measure which was not measured in any of the studies. Also, none of the studies reported which teeth were lost, and hence give no idea of the scheme of occlusal instability [68]. Finally, all the observational studies had a duration of under 10 years, which failed to address the chance of reverse causality [67]. Another recent systematic review pointed out that studies which researched associations between dementia and tooth loss for a period of ≥10 years have shown a decline in the effect of periodontal disease on cognitive deterioration as compared with the studies included in this review [34]. This could suggest reverse causality, since neurodegenerative changes leading to mild cognitive impairment and dementia are slow to develop over many years. Similar findings have been reported by other studies, showing that advanced cognitive impairment, as in cases of dementia and Alzheimer’s disease, lead to a disruption of oral hygiene procedures and a decline in periodontal health [69,70]. There is also a possibility that oral hygiene habits, smoking and alcohol consumption may have affected the outcomes reported in these studies [71].

Even with the many limitations, the strengths of this review are important to consider. Firstly, all the studies that were examined investigated a causal relationship between a decline in oral health as an exposure to the outcome of interest, that is, mild cognitive impairment. There has not been a systematic review that examined the association of mild cognitive impairment with tooth loss and periodontal disease in the same population, where all exposures and outcomes were clinically investigated and confirmed. Secondly, an overall large sample of the population from three different countries was examined in the studies, suggesting the global prevalence of the association of tooth loss and cognitive impairment. Thirdly, 75% of the selected studies investigated the impact of losing eight or more teeth on the development of mild cognitive impairment; it is important to note that loss of eight or more teeth in a dentition of 28 teeth (excluding the third molars) is important from the limited dental perspective [72,73]. Even though there were methodological heterogeneities among the studies, the subgroup meta-analysis conducted as part of the data synthesis helped to outline the risk of MCI separately against the severity of periodontal disease, tooth loss and both periodontal disease and tooth loss together in combination, due to the increased sample size.

Irrespective of the underlying mechanisms, a number of various studies indicated a high risk of cognitive impairment following loss of a higher number of teeth. The findings from this systematic review indicated that there is an increased risk of developing mild cognitive impairment in older adults when exposed to chronic periodontal disease and tooth loss, thus answering the review question. However, as the evidence collected was of low quality, the findings from this review also highlight the requirement to improve the design of longitudinal studies in the future, investigating the impact of tooth loss and periodontal disease on deterioration in cognitive function, with particular attention to s longer duration of follow-up of the cohort and the development of consistent validated methods to thoroughly assess periodontal disease, tooth loss and cognitive impairment status at baseline and follow-up. There is also a need for conducting such studies in underdeveloped and developing countries where the prevalence of periodontitis and tooth loss is higher. This should also facilitate research into exploring the underlying mechanisms and potential interventions to prevent or delay cognitive decline through oral health management. It also emphasized an urgent need for widespread oral health education and prevention programs for people aged 50 and above, who are generally at a higher risk of developing mild cognitive impairment and dementia [74]. Health care professionals all around the world need to be aware of this risk of an association between tooth loss and cognitive decline, and oral health should be prioritized for all individuals at risk of dementia, according to evidence-based dentistry [75]. Closely monitored preventive oral health care and targeted interventions to minimize tooth loss may provide new hopes for reducing the risk of dementia in the general population in future [1].

## 5. Conclusions

This systematic review demonstrated that there is a significantly higher risk of an association between mild cognitive impairment and both tooth loss and periodontal disease. However, the evidence in the available literature is insufficient to clarify the exact underlying mechanisms behind this association. There is a strong need for developing robust methodologies for future longitudinal studies with a longer duration and uniformity of the assessment methods in this field, which would help to elucidate role of periodontal pathogens and tooth loss as risk factors for neurocognitive disorders, thus helping to formulate and implement efficient and effective dementia prevention strategies. Clinically, more evidence is required to facilitate clear methods for oral health care professionals to identify at-risk individuals early, as they are well placed in the community, having regular contact with older adults.

## Figures and Tables

**Table 1:** Study characteristics.

Author and Publication Year	Kaye et al., [43]	Okamoto et al., [44]	Luo et al., [45]	Nilsson et al., [46]
**Population Description**	Male members of US Deparment of VA DLS receiving medical and dental care in private sector	Men and Women aged 65 and over who were residents of Nara prefecture	Immigrant Population from Hispanic/Latino population in 4 centres from US.	Older adults from Swedish National Study on Ageing and Care
**Location of Study**	Boston, Massachusetts	Japan	Newyork, Chicago, Miami and San Diego	Sweden –Blekinge
**Participants**				
**Number**	1231	3696	6377	775
**Age at baseline**	28–70 years	Over 65 years	50–74 years	Over 60 years
**Sex**	Male	Male & Female	Male & Female	Male & Female
**Withdrawals & Dropouts**	634 (51.5 %)	1361 (36.8%)	668 (10.5%)	N/A
**Study Design**	Prospective Observational	Prospective Observational	Longitudinal prospective cohort	cross –sectional
**Data collection in:**	8 Years	5 Years	ongoing since 2006	
**Start Date**	1993	2007	2006	
**End Date**	2001	2012	ongoing	2007–2009
**Methods to assess Periodontal Health:**	Number of teeth showing increase in PPD by ≥2 mm from baseline	CPI code (WHO probe) on 10 representative teeth in 6 segments of oral cavity (tooth: 11, 16, 17, 26, 27, 31, 36, 37, 46 & 47) codes 0–4. Highest code noted as maximum CPI code for individual	No/mild/moderate/severe disease based on interproximal attachment loss (PPD) and ABL according to CDC-AAP definition	Proportion of teeth with ≥5 mm PPD (from gingival margin to base of sulcus) on >30% of teeth & ABL (distance from CEJ to alveolar bone) > 4 mm on OPG at ≥30% of sites
	Number of teeth showing decrease in bone height by ≥40% from baseline			
**Methods to assess number of teeth:**	Number of teeth lost/decade of follow up	Remaining teeth categorised into 5 groups at baseline and follow up: 0/1–8/9–16/17–24/25–32	STL counted as 8 or more teeth lost at follow up compared to baseline	Number of teeth remaining categorised into 2 groups: 1–19/≥20
**Mild Cognitive Impairment accessed by:**	MMSE (MCI < 25)	MMSE (MMI ≥ 24)	Various neuropsychiatric battery tests including six item screener, verbal learning tests, word fluency, Digit symbol tests, trail making tests	MMSE (MCI < 25)
	Spacial copying task (MCI < 10)	Word recall test (MMI –0/1)		Clock test (MCI < 8)
		GDS score (MMI ≤ 5)		
**Conclusion**	Risk of cognitive decline in older men increases as more teeth are lost. Periodontal disease and caries (major reasons for tooth loss) are also related to cognitive decline.	Tooth loss predicts the development of Mild memory impairment in the elderly.	Significant tooth loss is a significant risk factor for mild cognitive impairment.	A history of periodontitis and tooth loss may be of importance for cognitive function among older adults.

Legenda: US—United States; VA—Veterans Affairs; DLS—Dental Longitudinal Study; CPI—Community Periodontal Index; WHO—World Health Organisation; PPD—Probing pocket depth; ABL—Alveolar bone loss; CEJ—Cemento enamel junction; OPG—Orthopantomogram; CDC—Centres for disease control and prevention; AAP—American academy of periodontology; STL—Significant tooth loss; MMSE—Mini Mental State Examination; GDS—Geriatric Depression Scale.

**Table 2:** Evaluation of studies’ quality using the Newcastle–Ottawa scale.

Criteria	Kaye et al. [43]	Okamoto et al. [44]	Nilsson et al. [46]	Luo et al. [45]
**Representativeness of the exposed cohort**	*		*	*
**Selection of the non-exposed cohort**	*		*	*
**Ascertainment of exposure (periodontitis and/or tooth loss)**	*	*		*
**Demonstration that the outcome (dementia/cognitive impairment) was not present at the start of the study**		*		*
**Comparability of cohorts according to the design and analysis**	*	*		*
**The study controls for additional factors (age, diet, smoking, education, socioeconomic factors, etc.)**	*	*	*	*
**Assessment of mild-cognitive impairment using validated assessment tools such as MMSE or the six-item screener test**	*		*	*
**Follow-up was long enough for the outcomes to occur (≥5 years)**	*	*	*	*
**Adequacy of follow-up of cohorts**	*			
**Total (maximum possible score = 9)**	8	5	5	8
	**High**	**Low**	**Moderate**	**High**

Legenda: (*) stands for present.

**Table 3:** Excluded studies and reasons.

Number	Study	Reason
1.	Han, J.H., Lee, H.J., Han, J.W., Suh, S.W., Lee, J.R., Byun, S., Kim, K.S., Kim, S.Y., Lee, J.T., Yoo, E., Chang, N.H., Kim, T.H. and Kim, K.W. (2020) ‘Loss of functional dentition is associated with cognitive impairment.’, *Journal of Alzheimer’s Disease,* 73(4), pp. 1313–1320. [13]	There was no consideration of periodontal disease as the cause for tooth loss; it studied functional occlusal units or teeth, not just remaining natural teeth. The exposure was different.
2.	Egashira, R., Umezaki, Y., Mizutani, S., Obata, T., Yamaguchi, M., Tamai, K., Yoshida, M., Makino, M. and Naito, T. (2021) ‘Relationship between cerebral atrophy and number of present teeth in elderly individuals with cognitive decline’, *Experimental gerontology,* 144, pp. 111189 Available at: 10.1016/j.exger.2020.111189. [48]	This study investigated the relationship of increased tooth loss with the severity of brain atrophy. No neuropshychological tests were employed. The exposure was different.
3.	Luo, J., Wu, B., Zhao, Q., Guo, Q., Meng, H., Yu, L., Zheng, L., Hong, Z. and Ding, D. (2015) ‘Association between tooth loss and cognitive function among 3063 Chinese older adults: A community-based study.’, *PLoS ONE,* 10(3) (pagination), pp. Arte Number: e0120986. ate of Pubaton: 24 Mar 2015. [49]	This study collected self-reported data on periodontal disease as the reason for tooth loss but did not report it as variables/exposure, as the authors did not consider the data to be reliable. The exposure was not fully reported.
4.	Matsuyama, Y., Fujiwara, T., Murayama, H., Machida, M., Inoue, S. and Shobugawa, Y. (2022) ‘Differences in brain volume by tooth loss and cognitive function in older Japanese adults.’, *American Journal of Geriatric Psychiatry,* 30(12), pp. 1271–1279. [50]	In this study, there was no consideration of periodontitis as a reason for tooth loss. The exposure was different.
5.	Panzarella, V., Mauceri, R., Baschi, R., Maniscalco, L., Campisi, G. and Monastero, R. (2022) ‘Oral health status in subjects with amnestic mild cognitive impairment and Alzheimer’s disease: Data from the Zabut Aging Project.’, *Journal of Alzheimer’s Disease,* 87(1), pp. 173–183. [51]	The exposure was amnestic mild cognitive impairment + Alzheimer’s disease, and the outcomes measured were oral health parameters such as caries and periodontal health. The exposure and outcome were different.
6.	Ganbaatar, U., Erdeneochir, U., Byambajav, P., Jadamba, T., Byambasukh, O. and Dagvajantsan, B. (2021) ‘Relationship of tooth loss to mild cognitive impairment among middle-aged Mongolians: Mon-timeline study.’, *Journal of the Neurological Sciences,* Conference: World Congress of Neurology (WCN 2021), pp. Arte Number: 119731. ate of Pubaton: Otober 2021. [27]	This study mainly concentrated on dental caries as the primary reason for increased tooth loss in the Mongolian middle-aged population. The exposure was different.
7.	Xu, S., Huang, X., Gong, Y. and Sun, J. (2021) ‘Association between tooth loss rate and risk of mild cognitive impairment in older adults: a population-based longitudinal study.’, *Aging,* 13(17), pp. 21599–21609. [31]	This study reported that a limitation was that they did not know the periodontal health of subjects, as no dental exam was carried out. Only self-reported number of teeth and information on denture use were collected. The exposure and the method used to assess exposure were different.
8.	Avlund, K., HolmPedersen, P., Morse, D.E., Viitanen, M. and Winblad, B. (2004) ‘Tooth loss and caries prevalence in very old Swedish people: The relationship to cognitive function and functional ability.’, *Gerodontology,* 21(1), pp. 17–26. [52]	In this study, the exposure was cognitive decline and outcome variable measured was teeth lost. The exposure and outcome were different.
9.	Naorungroj, S., Slade, G.D., Beck, J.D., Mosley, T.H., Gottesman, R.F., Alonso, A. and Heiss, G. (2013) ‘Cognitive decline and oral health in middle-aged adults in the ARIC study’, *Journal of Dental Research,* 92(9), pp. 795–801 Available at: 10.1177/0022034513497960. [53]	In this study, the exposure was cognitive decline, and the outcome variable measured was teeth lost and periodontitis. The exposure and outcome were different.

**Table 4:** Risk of cognitive decline in relation to periodontal disease’s progression: adjusted for age, gender and education.

Study	Sample Size	Age at Baseline	Main Exposure	Exposure Cut-Off Point	Pooled Ratio	95% Lower Limit	95% Upper Limit	*p*
Kaye et al. [43]	1231	28–70	Each additional tooth with ABL progression/decade	40% increase from baseline or teeth lost	1.03 (HR)	1.00	1.07	n/a
			Each additional tooth with PPD progression/decade	≥2 mm from baseline or teeth lost	1.04 (HR)	1.01	1.09	
Okamoto et al. [44]	3696	>65	CPI codes 0–4	Code 4	1.03 (AOR)	0.73	1.45	0.871
Nilsson et al. [46]	775	>60	Distance from CEJ to bone level	≥4 mm on ≥30% sites	2.7 (AOR)	1.2	5.9	0.013
Luo et al. [45]	6377	>50	% of teeth with attachment loss, and % of teeth with increased PPD	≥6 mm on ≥2 sites, and ≥5 mm on ≥2 interproximal sites	1.02 (AOR)	0.67	1.54	0.93

**Table 5:** Risk of cognitive decline in relation to tooth loss, adjusted for age, gender and education.

Study	Sample Size	Age at Baseline	Exposure Cut-Off Point	Pooled Ratio	95% Lower Limit	95% Upper Limit	*p*
Kaye et al. [43]	1231	28–70	Each additional tooth lost/decade	1.09 (HR)	1.01	1.18	n/a
Okamoto et al. [44]	3696	>65	Each tooth lost at follow-up	1.01 (AOR)	1.00	1.03	0.051
			Edentulous	2.32 (AOR)	1.44	3.74	0.001
Nilsson et al. [46]	775	>60	1–19 teeth	2.0 (AOR)	1.1	3.6	0.03
Luo et al. [45]	6377	>50	≥8 teeth	1.46 (AOR)	1.09	1.95	0.01

## Data Availability

Data are contained within the article.
